# Electrospun Polyhydroxybutyrate and Poly(L-lactide-*co*-**ε**-caprolactone) Composites as Nanofibrous Scaffolds

**DOI:** 10.1155/2014/741408

**Published:** 2014-05-08

**Authors:** Donraporn Daranarong, Rodman T. H. Chan, Nico S. Wanandy, Robert Molloy, Winita Punyodom, L. John R. Foster

**Affiliations:** ^1^Biomedical Polymers Technology Unit, Department of Chemistry, Faculty of Science, Chiang Mai University, Chiang Mai 50200, Thailand; ^2^Bio/Polymer Research Group, School of Biotechnology & Biomolecular Sciences, University of New South Wales, Sydney, NSW 2052, Australia; ^3^Materials Science Research Center, Faculty of Science, Chiang Mai University, Chiang Mai 50200, Thailand

## Abstract

Electrospinning can produce nanofibrous scaffolds that mimic the architecture of the extracellular matrix and support cell attachment for tissue engineering applications. In this study, fibrous membranes of polyhydroxybutyrate (PHB) with various loadings of poly(L-lactide-co-*ε*-caprolactone) (PLCL) were successfully prepared by electrospinning. In comparison to PLCL scaffolds, PLCL blends with PHB exhibited more irregular fibre diameter distributions and higher average fibre diameters but there were no significant differences in pore size. PLCL/PHB scaffolds were more hydrophilic (<120°) with significantly reduced tensile strength (ca. 1 MPa) compared to PLCL scaffolds (150.9 ± 2.8° and 5.8 ± 0.5 MPa). Increasing PLCL loading in PHB/PLCL scaffolds significantly increased the extension at break, (4–6-fold). PLCL/PHB scaffolds supported greater adhesion and proliferation of olfactory ensheathing cells (OECs) than those exhibiting asynchronous growth on culture plates. Mitochondrial activity of cells cultivated on the electrospun blended membranes was enhanced compared to those grown on PLCL and PHB scaffolds (212, 179, and 153%, resp.). Analysis showed that PLCL/PHB nanofibrous membranes promoted cell cycle progression and reduced the onset of necrosis. Thus, electrospun PLCL/PHB composites promoted adhesion and proliferation of OECs when compared to their individual PLCL and PHB components suggesting potential in the repair and engineering of nerve tissue.

## 1. Introduction

Tissue engineering is an interdisciplinary field that uses the principles of engineering and life sciences to the development of biological substitutes that restore, maintain, and improve the function of damaged tissues and organs [1, 2]. A number of studies have focused on the development and design of biomaterial scaffolds for permanent tissue replacements. Biomaterials designed for such scaffolds should be biocompatible and biodegradable and should not trigger any severe immune responses from the host [3]. Polyesters, such as poly(lactic acid) (PLA), poly(*ε*-caprolactone) (PCL), polyhydroxybutyrate (PHB), and their copolymers closely fulfill these criteria and are potential biomaterials for tissue engineering [4–6].

A common approach in scaffold engineering is to mimic the architecture of the natural extracellular matrix (ECM). Electrospinning has been used to produce nanofibrous scaffolds consisting of high surface area to volume ratios and various pore sizes that mimic the tissue ECM morphology and support cell attachment, proliferation, and differentiation [7–9]. A number of polymers have been successfully fabricated as electrospun scaffolds, including PHB, PLA, PCL, and their copolymers [4, 5].

**Figure 1:**
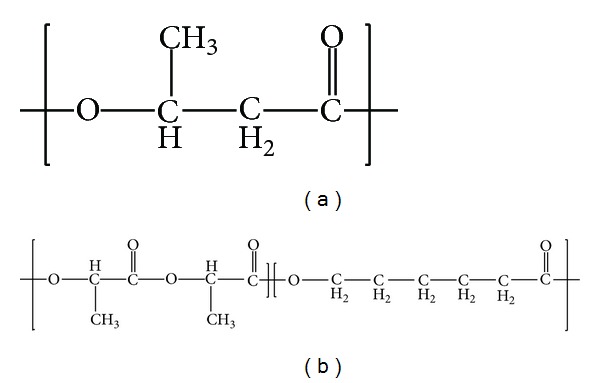
Chemical structure of polymers used in this study, (a) polyhydroxybutyrate (PHB) and (b) poly(L-lactide-co-*ε*-caprolactone) (PLCL).

Polyhydroxybutyrate (PHB) is biodegradable aliphatic polyester synthesized by a wide variety of bacteria from renewable carbon sources (Figure 1(a)). The monomeric component of PHB, 3-hydroxybutyrc acid (3-HBA), is a stable analyte in human sera and microbial HBA is recognized by the mammalian system [10]. Consequently, PHB has been explored as a biomaterial for medical devices and is now FDA approved. Furthermore, Xiao et al. report that PHB has the potential as a neural protective agent and reduces the number of apoptotic mouse glial cells when compared to those exhibiting asynchronous growth [11]. However, PHB has a comparatively high crystallinity that results in a brittle nature and a relatively long degradation time under physiological conditions, limiting its potential applications [12, 13]. A number of studies have attempted to modify the material properties of PHB through blending [14–16]. Chan et al. report that blending PHB with a variety of PEGs significantly reduced PHB crystallinity and increased the extension at break of these composite films [15]. Changes to the surface morphology and hydrophilicity as a consequence of the blending were also found to increase cell attachment and proliferation of neural-associated olfactory ensheathing cells (OECs). Similarly, Sombatmankhong et al. reported that a scaffold consisting of a 50 : 50 (w/w) blend of PHB with poly(3-hydroxybutyrate-co-3-hydroxyvalerate) (PHBV) exhibited comparatively better support for the attachment and proliferation of human osteoblasts and mouse fibroblasts [16]. Films of PHB blended with poly(L-lactide-co-*ε*-caprolactone), PLCL, have potential in supporting cardiovascular, cartilage, and nerve regeneration [17–19]. PLCL is a synthetic, highly elastomeric, biodegradable, and nontoxic copolymer (Figure 1(b)). The PLCL copolymer is composed of PLA and PCL which have been approved by the Food and Drug Administration (FDA) in the USA and have been extensively studied for biomedical applications including controlled-release drug delivery systems, monofilament surgical sutures, and absorbable nerve guides [17–19]. Thus, PLCL is an interesting candidate for blending with PHB to potentially obtain a scaffold with improved physical and mechanical characteristics, as well as promote cell adhesion, proliferation, and differentiation.

Biomaterial chemistry is known to influence cell growth and more recently has been shown to affect their progression through the cell cycle [20]. The development, proliferation, and differentiation of eukaryotic cells are regulated through their cell cycle. Progression through this cycle is controlled by the appropriate and judicious activation and inactivation of cell cycle related proteins [21]. Tissue engineering relies upon the seeding of biomaterial scaffolds with stem cells and requires two cellular processes to occur concurrently. Cells must spread and populate the scaffold through cell proliferation, whereby cells cycle through a “resting” phase, (G0/G1), DNA synthesis (S), mitosis (M), and a gap between synthesis and mitosis phases (G2). Typically, the G0/G1 phase is shortened when cells move from a nonproliferative to a proliferative state, while the S, G2, and M phases remain relatively constant. While the biocompatibility of a variety of nanofibrous scaffolds has been reported for various cell lineages, including endothelial cells (ECs), mesenchymal stem cells, mouse fibroblasts (L929), and Schwann cells (RT4-D6P2T), the influence of the scaffolds on cell cycle progression has not been reported [6, 8, 22, 23].

In this study, various blends of PLCL and PHB were explored for electrospinning into nanofibrous scaffolds. Surface properties of these scaffolds were characterized and their biocompatibility was assessed by analysing the response of olfactory ensheathing cells (OECs) in terms of cell proliferation and health. OECs are a type of glial cell vital in promoting the regeneration of nascent neurons in the olfactory system [24]. The olfactory system promotes axonal outgrowth from the peripheral nervous system (PNS) into the central nervous system (CNS) owing to the presence of both peripheral and central tissues [24, 25]. Following trauma, OECs leave their quiescent stage and reenter the cell cycle to proliferate and regenerate tissue. Eventually, submucosal-derived OECs attain a stage where they repopulate to form the neurons of the olfactory epithelium [26]. Thus, OECs are an attractive cell lineage for the regeneration and engineering of nerve tissue and are reported to promote functional recovery at the site of spinal cord injury (SCI) [24, 25].

## 2. Materials and Methods 

### 2.1. Materials and Reagents

A statistical copolymer of poly(L-lactide-co-*ε*-caprolactone), PLCL 67 : 33 (mole %), was synthesized by ring-opening bulk polymerization (ROP) at 120°C for 72 hours using SnOct_2_ as the initiating system [27, 28]. Polyhydroxybutyrate (PHB) of natural origin and trypsin was purchased from Sigma Aldrich (Sydney, Australia). Analytical grade chloroform and dimethyl sulfoxide (DMSO) were purchased from Univar (Seven Hills, Australia). Mammalian cell growth medium, foetal bovine serum (FBS), and penicillin/streptococcus antibiotic were obtained from Gibco-Invitrogen (Sydney, Australia). OECs were routinely cultured in Dulbecco's Modified Eagle's Medium (DMEM/F12) supplemented with 10% FBS purchased from Lonza (USA).

### 2.2. Fabrication of PLCL/PHB Nanofibrous Scaffolds

PLCL and PHB in ratios 100 : 0, 75 : 25, 50 : 50, 25 : 75, and 0 : 100 (% w/w) were dissolved in a mixed solvent system of chloroform : dimethylformamide (DMF) in a ratio of 7 : 3 (% v/v) at a concentration of 6 (% w/v). Polymer solutions were electrospun at 15 kV with a needle to collector distance of 15 cm for 30 min at 40°C and 30% relative humidity (rH) with a flow rate of 4 mL h^−1^, using an IME Climate-Controlled Electrospinning Apparatus equipped with a plate collector module (models: EC-CLI, EM-PPC and EM-CAX, MTS technologies, The Netherlands). Fibre samples were then dried under vacuum (25°C, 12 h), removed, and allowed to stand in a clean, biosafety cabinet to an atmospherically equibrillated weight (25°C, ~12 h). Spinning of both PLCL and PHB components into fibres was confirmed by Fourier transform infrared spectroscopy (FTIR) as per [8]. Morphology of the electrospun samples was examined by SEM (FEI Quanta 200 ESEM, Japan) at an acceleration voltage of 15 kV. Diameters and pore sizes were analysed from a random sampling of 50 fibres per SEM image using image analysis software (ImageJ, National Institutes of Health, USA).

### 2.3. Scaffold Characterization

Scaffold samples (30 × 15 mm) were fixed using pneumatic grips to a calibrated tensile testing instrument (Instron-5543, Norwood, MA, USA) and slowly moved apart (20 mm min^−1^, 22°C, 30% rH). The maximum load, tensile strength, and extension at break were calculated using Bluehill Computer Software (Norwood, MA, USA). Means from at least ten samples were determined (*n* = 10) [15].

Scaffold hydrophilicity was measured through water contact angles using a sessile drop contact angle meter (KSV Cam 200, Espoo, Finland) [29]. Water droplets were placed on samples of electrospun scaffolds (40 × 30 mm) and their contact angles with the scaffolds were measured using KSV instrument software (22°C, rH 30%). Means of five readings were calculated for each sample (*n* = 5).

### 2.4. Cell Studies

Murine olfactory ensheathing cells (OECs) were cultivated in a medium consisting of DMEM, 10% FBS, 250 units penicillin, 250 *μ*g mL^−1^ streptomycin and 1 *μ*g mL^−1^, and fungizone-amphotericin B in T-75 tissue culture flasks (37°C, 5% CO_2_) [24]. At 70% confluence, OECs were removed using trypsin (2.5%) and cell populations of approximately 3 × 10^3^ cells mL^−1^ used to seed PLCL, PHB, and PLCL/PHB (50 : 50) composite fibre scaffolds (1 cm diameter) in 24-well plates. Samples were sacrificed at periodic intervals and rinsed twice with 10 mL of PBS; trypsin (2 mL, 2.5%) was subsequently added before incubation (37°C, 2 mins). Cell viability was then calculated using the trypan blue exclusion technique and a haemocytometer. Samples were conducted in triplicate (*n* = 3).

### 2.5. Microscopy

Scaffolds were rinsed twice with PBS (1%) and fixed in 2.5% glutaraldehyde in 0.1 M PBS (pH 7.2, 4 h, 22°C). Scaffolds were subsequently subjected to multiple washings (×4, 5 min each) with PBS buffer; after the final wash, scaffolds were dehydrated in a series of ethanol washes (30, 50, 70, 80, 90, 95, and 100%) for 10 min each, before critical point drying using liquid carbon dioxide. Specimens were then mounted on aluminium stubs and sputter coated with gold before examining using scanning electron microscopy (FEI Quanta 200 ESEM, Japan).

### 2.6. Mitochondrial Activity and Membrane Integrity

A fixed number (3 × 10^3^ cells) of OECs cultured in DMEM-10% FBS medium were plated into 96-well plates containing the polymer scaffolds and incubated for 168 h, (37°C, 5% CO_2_). Mitochondrial activities in the OEC populations were assessed using a CellTiter 96 AQueous one solution cell proliferation assay, where 30 *μ*L of MTS (3-(4,5-dimethylthiazol-2-yl)-2,5-diphenyltetrazolium bromide) solution was added to each well and incubated for a further 4 hours (37°C, 5% CO_2_), while membrane integrity was assessed through leakage of lactate dehydrogenase (LDH). Samples of lysis solution (10 *μ*L) were added to 5 of the wells 45 min prior to the endpoint and these served as positive controls. The LDH assay plate was subsequently centrifuged (5 mins, 250 g, 22°C, rH 30%) and 50 *μ*L samples of the supernatants were transferred to a sterile 96 well-plate with 100 *μ*L of LDH mixture being added to each well, before incubating in the dark (30 min, 37°C, 5% CO_2_). MTS and LDH analyses were performed at 490/690 and 490/650 nm wavelengths, respectively, using a microtitre plate spectrophotometer. Means of 5 samples were determined (*n* = 5) [30, 31].

### 2.7. Cell Cycle and Apoptosis

OECs (3 × 10^3^ cells) were cultured in DMEM medium containing 10% FBS in sterile 24 well-plates coated with scaffolds while controls in the absence of biomaterials were simultaneously conducted under the same conditions. Controls performed to validate the acquired data included (1) cells in 10% FBS, (2) serum-deprived cells, (3) cells synchronized for 24 hours in medium containing 10% FBS and 1 *μ*g mL^−1^ aphidicolin, and (4) 2 *μ*M nocodazole. Similar controls, but with the absence of (3), were used to determine the apoptotic indices associated with the externalisation of phosphatidylserine (PS) [30, 32]. After 5 days of incubation, cells were removed using trypsin then excess FBS media were added to neutralise the trypsin reaction. Cells were subsequently washed with DPBS and fixed with ice-cold ethanol (70%, −20°C, 12 h) before centrifuging (300 g, 5 min). The cell pellet was resuspended in propidium iodide (PI) staining solution (0.1% v/v Triton X-100, 0.2 mg/mL RNAase, and 20 *μ*g/mL PI in DPBS). Cells were subsequently incubated in the dark (22°C, 30 mins) before analysing using a Tali Image-Based Cytometer (Invitrogen, USA).

For analysis of early stage apoptosis, cell samples were washed with DPBS and suspended in Annexin binding buffer and Annexin V Alexa Fluor 488 (Invitrogen, USA) prior to incubation in the dark (22°C, 30 mins). Cells were then extracted through centrifugation (300 g, 5 mins) and resuspended in Annexin binding buffer and Tali PI solution before briefly incubating in the dark (22°C, 5 mins). The Tali Image-Based Cytometer was then used to measure the apoptotic indices.

### 2.8. Statistical Analysis

All data was statistically evaluated using the two-way ANOVA analysis and Bonferroni post-test (significance level < 0.05).

## 3. Results and Discussion

### 3.1. Polymer Characterization

**Figure 2:**
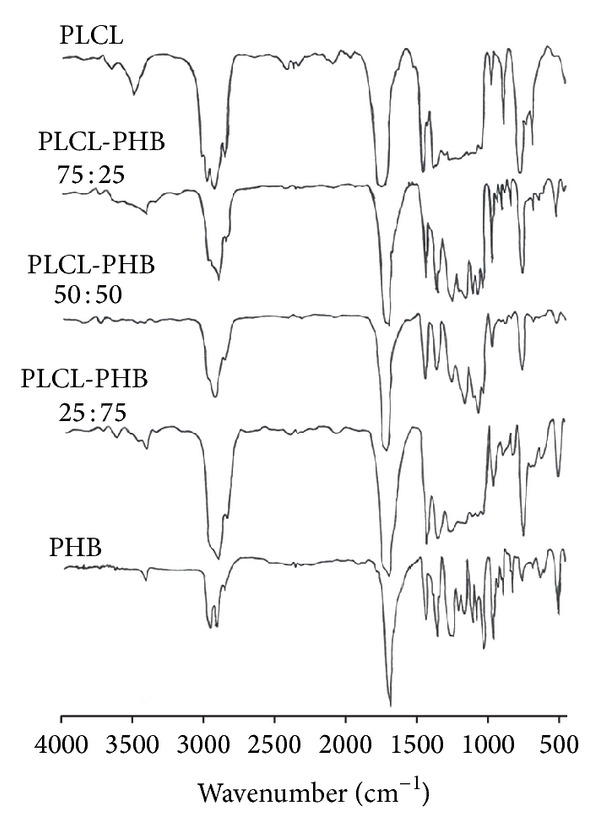
FTIR spectra of solubilised electrospun scaffolds fabricated from PLCL, PHB, and blends of PLCL/PHB with different loadings (% w/w).

Mixed solutions of PHB and PLCL were electrospun into nanofibrous scaffolds, samples were subsequently resolubilized and analysed using FTIR to confirm the presence of both PHB and PLCL in the fibres. The FTIR spectrum in Figure 2 shows a number of peaks assigned to bonds representing both the PLCL and PHB components (Figure 2, Table 1). The C–O stretching at 1056 cm^−1^ and O–H bending at 1380 cm^−1^ are consistent with the presence of PLCL. The peak at 1753 cm^−1^ corresponds to C=O stretching of the PHB ester group present in the molecular chain of a highly ordered crystalline structure, while the peak at 1273 cm^−1^ corresponds to the –CH group [33]. Thus, electrospun PLCL/PHB fibres showed major peaks consistent with both PLCL and PHB components.

### 3.2. Morphology of Electrospun Scaffolds

**Figure 3:**
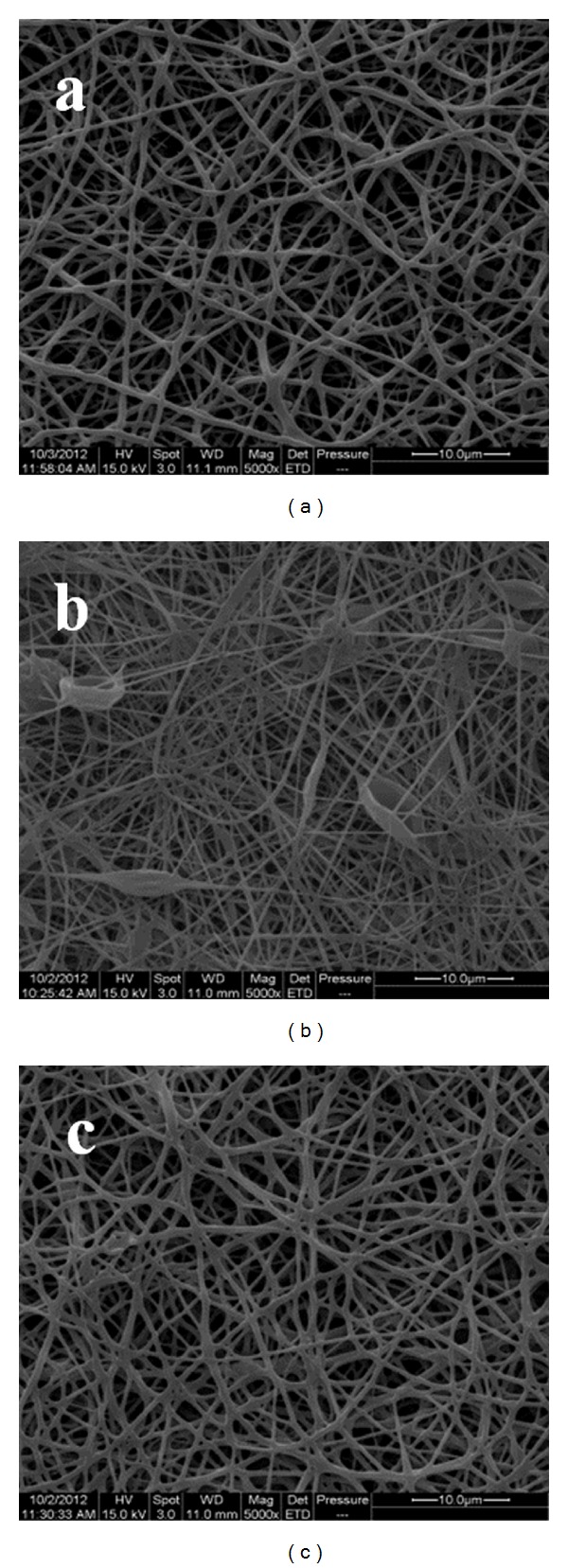
SEMS of electrospun nanofibrous matrices of (a) PHB, (b) PLCL, and (c) a 50 : 50 (% w/w) blend of PHB/PLCL (Magn: ×5 k, bar = 10 *μ*m).

**Figure 4:**
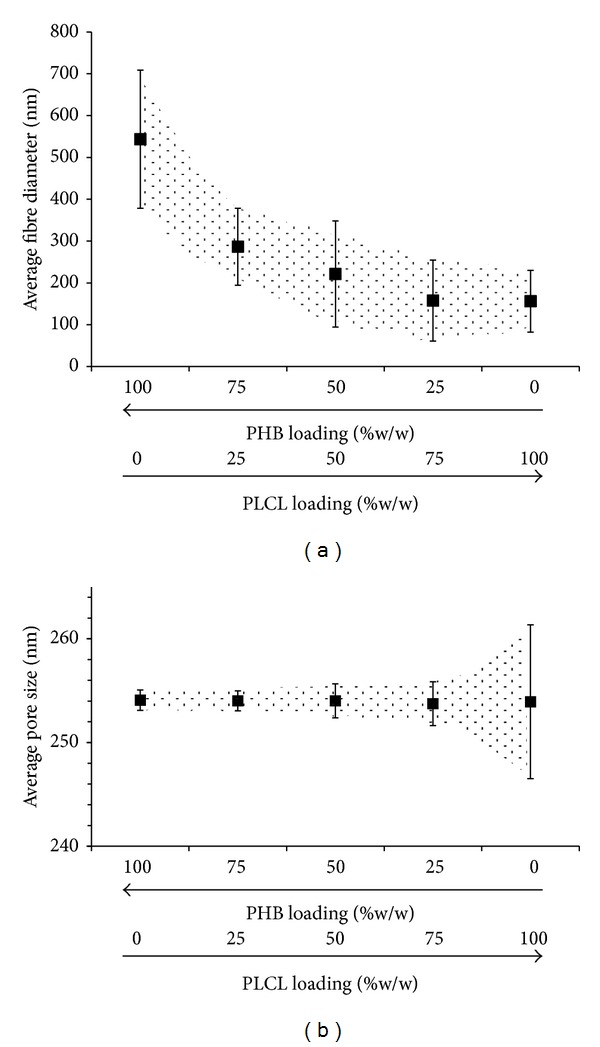
Changes in average (a) fibre diameter (*μ*m) and (b) pore size (*μ*m) of electrospun PHB scaffolds and their blends with PLCL (% w/w, *n* = 50); bars and shaded regions show variations in the distribution.

Electrospinning can produce nanofibrous scaffolds that mimic the architecture of the extracellular matrix (ECM) and consequently promote cell growth, proliferation, and differentiation [7–9]. In this study, the respective loadings of PLCL and PHB had a direct relation to electrospun fibre morphology. The nanofiber morphologies of electrospun mats appeared as nonwoven fibres with interconnected pores and some beads in the PLCL mat (Figure 3(b)). Electrospun PHB fibres showed different morphologies when compared to their centrifugally gel-spun counterparts which appeared like cotton wool and had individual fibres with a diameter range between 1 and 15 *μ*m [34]. SEMs of electrospun PHB revealed a matrix of fibres with a mean diameter of 543.5 ± 165.1 nm (Figure 3(a)). In contrast, PLCL fibres electrospun under the same conditions showed a significantly smaller mean diameter of 156.1 ± 73.9 nm (Figure 3(b)). Blending PHB with 25, 50, and 75% (w/w) PLCL resulted in a gradual decrease in the mean diameters of the fibres, 286.3 ± 91.9, 221.4 ± 127.1, and 157.9 ± 96.9 nm, respectively (Figure 4(a)). The 25% (w/w) loading of PHB in the PLCL fibrous scaffold showed no significant difference in its mean diameter compared to the PLCL scaffold. Despite these changes in diameter, there were no significant changes in the average pore size which remained about 254 ± 3 nm, although the distribution of pore sizes appeared to increase with PLCL loading (Figure 4(b)).

**Figure 5:**
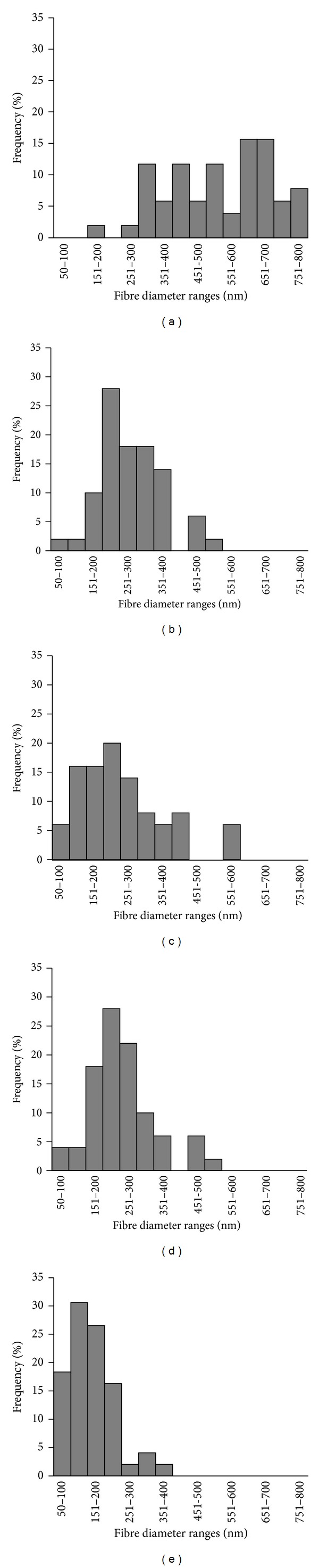
Changes in the distribution of diameter of the fibre (*μ*m) for electrospun scaffolds of (a) PHB; (b) PHB/PLCL at 75 : 25 (%, w/w); (c) PHB/PLCL at 50 : 50 (%, w/w); (d) PHB/PLCL at 25 : 75 (%, w/w); and (e) PLCL.

Figure 5 reveals that blending PHB with PLCL also changed the diameter distributions. While the PHB scaffold consisted of fibres exhibiting a comparatively wide distribution of diameters, ranging from 151 to 800 nm with less than 15% frequency for each diameter (Figure 5(a)), the PLCL showed a narrow distribution range from 50 to 400 nm but with 55% of the fibres possessing a diameter between 100 and 200 nm (Figure 5(e)). Blending the PHB with 25% (w/w) PLCL shifted the fibre diameters distribution from 50 to 600 nm but with approximately 85% of the fibres exhibiting diameters between 150 and 400 nm (Figure 5(b)). As the PLCL loading increased the shift towards smaller diameters, such that the electrospun scaffold with equal loadings of PHB and PLCL (50 : 50% w/w) consisted of fibres with a comparatively narrower diameter range (from 50 to 400 nm, Figure 5(c)). In contrast to the PHB scaffolds with 25% (w/w), PLCL loading which had only 4% of its fibres possessing diameters below 200 nm, the scaffold with 50% (w/w) PLCL exhibited approximately 21% below 200 nm. Electrospun scaffolds with a greater loading of PLCL (75% w/w) had approximately 69% of its fibres with diameters between 150 and 350 nm compared to the PLCL scaffolds which had 93% of their fibres possessing diameters below 250 nm (Figures 5(d) and 5(e)). It is clear that nanofiber diameters and their distributions in electrospun PHB scaffolds could be easily modified by blending with PLCL where the greater loading of PLCL favoured smaller diameters and a more homogenous distribution.

**Figure 6:**
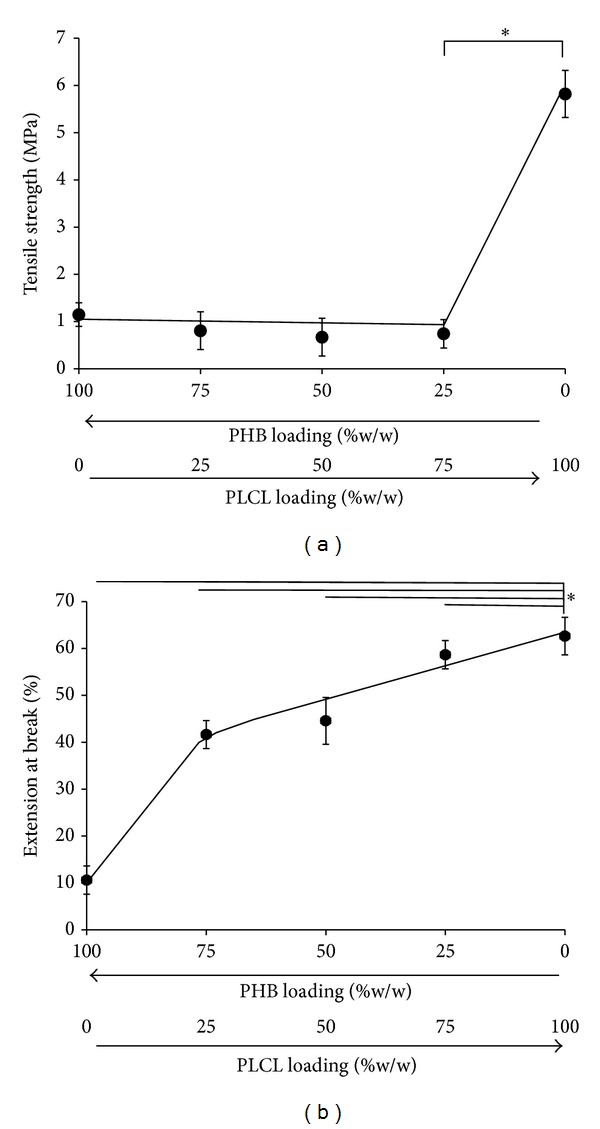
Changes in (a) tensile strength (MPa) and (b) extension at break (%) for PHB electrospun scaffolds and its blends with PLCL (% w/w), (*significant *P* < 0.05, *n* = 5).

Blending is recognized as a cost-effective technique for the manipulation of material and physiochemical properties of polymeric biomaterials. Electrospun PHB scaffolds exhibited a tensile strength of approximately 1.2 ± 0.2 MPa, blending PHB with PLCL had no apparent effect on tensile strength, but the PLCL scaffolds had a tensile strength of 5.8 + 0.5 MPa (Figure 6(a)). This suggests that the crystallization of PHB in the blend was unaffected by the PLCL. However, the PHB scaffolds exhibited an extension to break of approximately 10.6 ± 1.4% which is higher than values for its solvent cast films (2.5 ± 1.8%) and a consequence of its comparatively high crystallinity [15]. Blending PHB with PLCL significantly reduced the brittleness of the electrospun fibres and significantly increased the extension to break PHB scaffolds with 25% (w/w) PLCL increased to 41.6 ± 0.8%. This trend continued as the PHB composition in the scaffolds was reduced until an extension of 62.6 ± 2.5% was determined for scaffolds consisting of PLCL only (Figure 6(b), *P* < 0.005). These improvements may be mainly attributed to the finely dispersed PHB crystals acting as a filler to enhance the properties of the PLCL matrix and the possible interaction between PHB and PLCL causing strong interfacial bonding. Moreover, the apparent properties of the fibrous membranes depended on the degree of fibre orientation, that is, the macromolecular design. In this research, the blended electrospun membranes became more flexible as the amorphous PLCL content increased due to a decrease of the overall crystallinity of the fibrous membranes.

**Figure 7:**
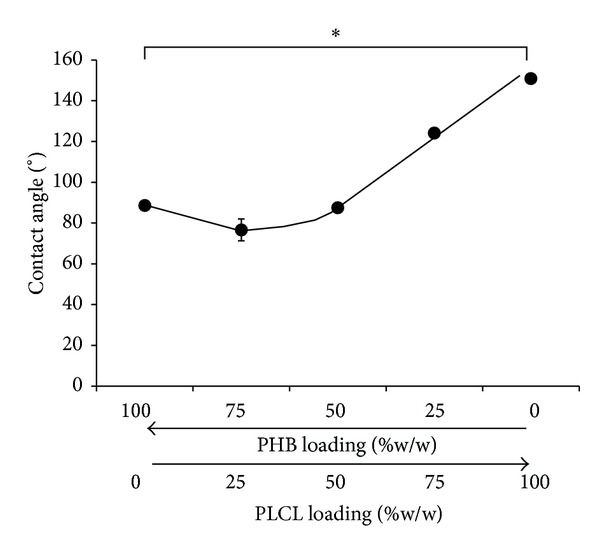
Changes in water contact angle of electrospun PHB scaffolds and their blends with PLCL (% w/w), (* = significance *P* < 0.05, *n* = 5).

Hydrophobicity of biomaterials as well as surface morphology of their devices is known to influence cellular adhesion and proliferation [35]. In this study, PHB electrospun scaffolds exhibited a water contact angle of 88.6 ± 0.5° while the PLCL scaffolds were apparently more hydrophobic, with a mean water contact angle of 150.9 ± 2.8° (Figure 7). However, these values may have been influenced by the presence of pores under the water droplet and the various fibre morphologies [36]. Blending PHB with 25% (w/w) PLCL showed no apparent change, but further increases in PLCL increased the hydrophobicity of the nanofibrous scaffolds in a linear fashion (Figure 7). It is clear, that blending PHB with PLCL can significantly influence the morphology and hydrophobicity of their electrospun scaffolds, which may have consequences for cell attachment and proliferation.

### 3.3. Biocompatibility of Electrospun Matrices

**Figure 8:**
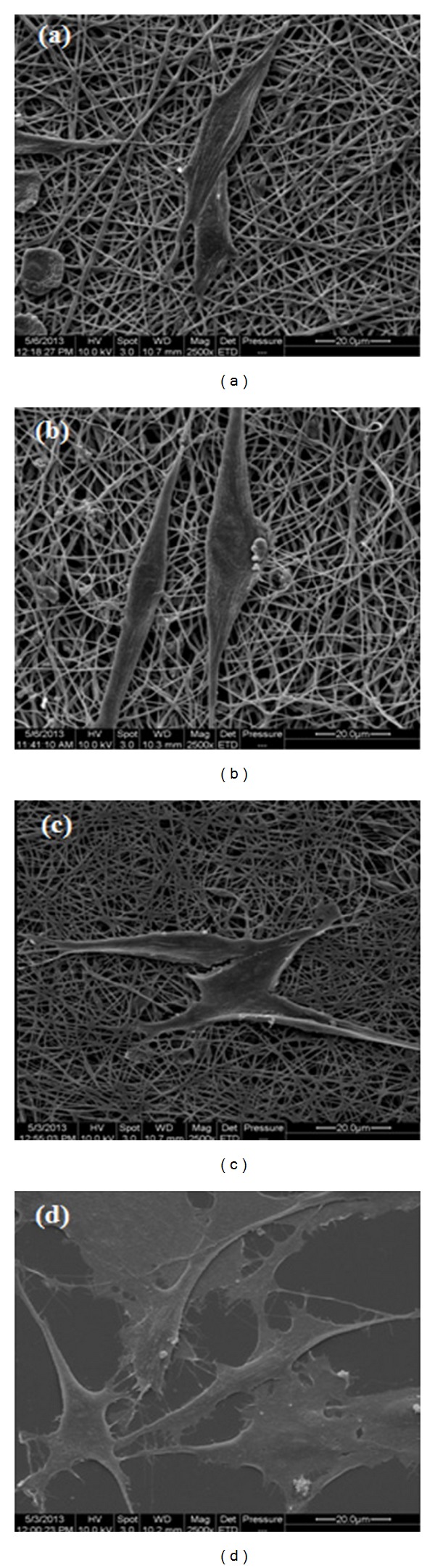
SEMs illustrating morphology of olfactory ensheathing cells attached to (a) electrospun PLCL, (b) PHB, (c) PLCL/PHB (50 : 50% w/w) scaffolds, and (d) tissue culture slide (Magn. ×2.5 k, Bar = 20 *μ*m).

To investigate the biocompatibility of the nanofibrous scaffolds, adult OECs were cultivated on the PLCL, PHB, and PLCL/PHB (50 : 50% w/w) electrospun membranes. OECs attached onto the tissue culture glass control displayed a healthy, flat appearance with many filopodial extensions (Figure 8). These filopodia play an important role in neuron regeneration and are the first step in growth cone formation [37]. Similarly, OECs cultivated on all the biomaterial scaffolds also displayed a similar spindle-like morphology but appeared to have more filopodial extensions when compared to the control (Figure 8).

**Figure 9:**
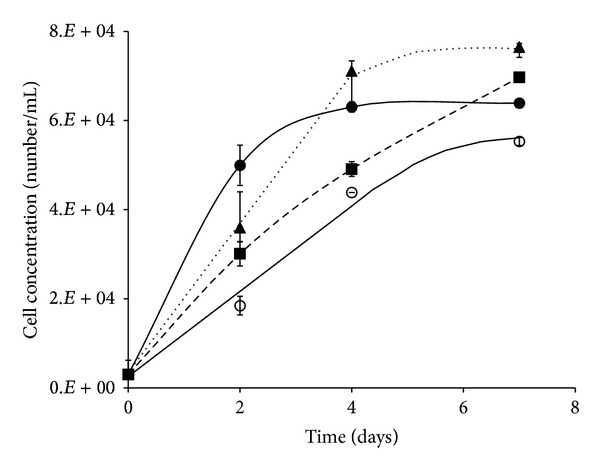
Changes in the growth of olfactory ensheathing cells (cells/mL) when cultivated on PLCL (■), PHB (●), and PLCL/PHB (▲) electrospun scaffolds; tissue culture plate (⚪), (*n* = 5).

Xu et al. have reported that an electrospun PLCL mat supported human coronary artery smooth muscle cell (SMC) and endothelial cell (EC) attachment and proliferation. When seeded on this scaffold, these cell lineages tended to maintain their phenotypic shape, they were also found to migrate through the pores of the nanofibrous scaffold [38]. In this study, OECs grew readily in a cell culture dish with a linear rate of approximately 3.29 × 10^2^ cells/mL/h. OEC proliferation on the PLCL scaffolds showed a similar linear trend but with a significantly greater rate of 4.15 × 10^2^ cells/mL/h (Figure 9). In contrast, OECs adhered more readily to the electrospun PHB scaffolds within the first 48 hours of incubation, with a rate of approximately 10.41 × 10^2^ cells/mL/h before gradually plateauing at 63.7 × 10^3^ cells/mL after 96 hours. OECs cultivated on scaffolds produced using an equal mix of the 2 polymers displayed a growth trend with characteristics similar to those cultivated on both polymers individually (Figure 9). Thus, an initial linear rate of 7.41 × 10^2^ cells/mL/h was observed, a reduction compared to growth rate on PHB, but was maintained for longer, eventually attaining approximately 71.1 × 10^3^ cells/mL after 96 hours (Figure 9). Therefore, in this study, cell adhesion and proliferation on PHB electrospun scaffolds were tempered by blending with PLCL, which is consistent with the significant increase in hydrophobicity. However, growth on the PLCL/PHB composite fibres was maintained for longer when compared to OEC growth on the PHB scaffold (Figure 9).

**Figure 10:**
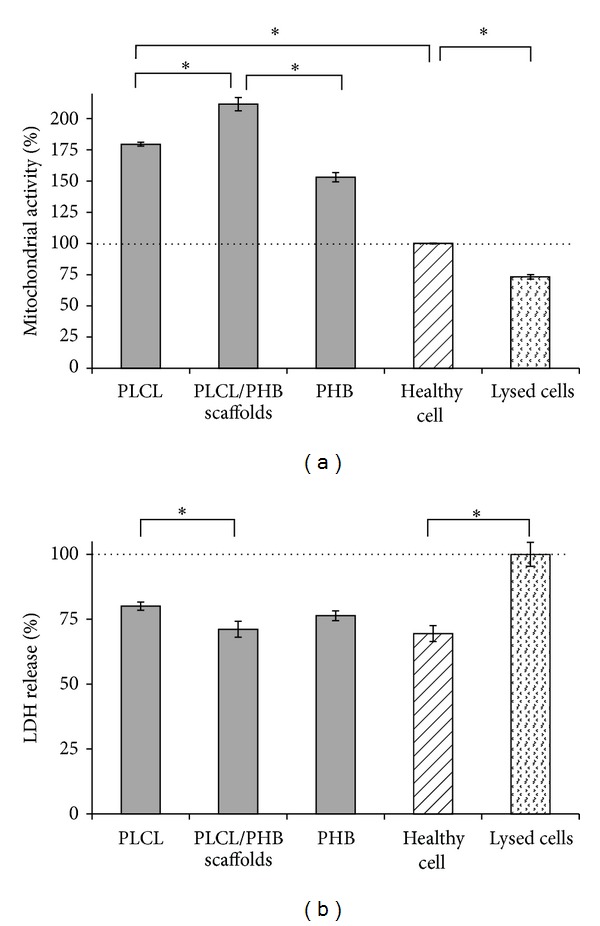
Relative health of OECs cultivated on electrospun scaffolds of PLCL, PHB, and PLC/PHB (50 : 50 w/w), measured by (a) mitochondrial activity (MTS assay) with cells in asynchronous growth assigned 100% and (b) membrane integrity (LDH assay) with lysed cells assigned 100% (*significant *P* < 0.05, *n* = 5).

The health of OECs on the nanofibrous scaffolds were evaluated by their mitochondrial activity and membrane integrity [30, 32].  Figure 10(a) shows that after 7 days of cultivation, cell populations on the electrospun fibres possessed significantly greater mitochondrial activities than OECs exhibiting asynchronous growth. After 7 days of incubation, OECs cultivated on PHB scaffolds showed an enhanced mitochondrial activity, 53 ± 4% above that of the OECs in asynchronous growth (allocated as 100%). In contrast, mitochondrial activity for OECs cultivated on the PLCL scaffold was 79 ± 2% greater. Consistent with the cell proliferation data, OECs cultivated on the blend of PHB and PLCL exhibited a significant 112 ± 5% increase (Figure 10(a)).

Membrane leakage of LDH in the medium provides an indication of cellular cytotoxicity [31]. In this study, the concentration of LDH released from OECs on the electrospun scaffolds showed no significant difference when compared with healthy cells (*P* > 0.05, Figure 10(b)). All the samples demonstrated a significantly lower LDH activity than the control of lysed cells (allocated 100%, *P* < 0.05, Figure 10(b)). These results suggest that the electrospun scaffolds were not cytotoxic to OECs, and this is consistent with the FDA status of these polymers, but actually promoted cell adhesion and proliferation.

**Figure 11:**
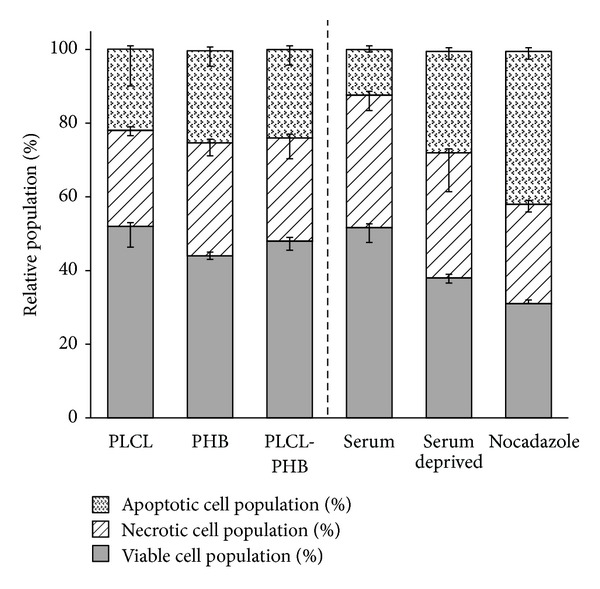
Relative health of OECs cultivated on electrospun scaffolds of PLCL, PHB, and PLC/PHB (50 : 50 w/w), expressed as apoptotic, necrotic, and viable populations (%) relative to cells in asynchronous growth; controls of cells in serum deprived media and with the addition of nocodazole used to validate the experiment (*significant *P* < 0.05, *n* = 5).

To further examine the viability of cells cultivated on the electrospun scaffolds, an Annexin V assay was used to detect early stage apoptosis, an important consideration in understanding programmed cell death [39]. Annexin V is a calcium-dependent phospholipid binding protein that binds to the phospholipid phosphatidylserine (PS). Most mammalian cells have PS within the plasma membrane which is externalized once apoptosis is initiated; it is subsequently detected on the cell surface by staining with an Annexin V FITC (fluorescein isothiocyanate) conjugate of high affinity [40]. OECs cultivated on the scaffolds showed similar distributions of viable cells to those in asynchronous growth (ca. 52 ± 4%, Figure 11). However, while 12 ± 1% of OECs cultivated in the absence of biomaterials showed spontaneous apoptosis, approximately 23 ± 4% of cells grown on the electrospun scaffolds were apoptotic. However, this increase in the proportion of apoptotic cells was apparently primarily due to a reduction in the proportion of necrotic cells, approximately 28 ± 3% for OECs cultivated on the scaffolds compared to 36 ± 3% for cells in asynchronous growth (Figure 11). Thus, the results suggest that the nanofibrous scaffolds promoted cell health by helping to prevent necrosis.

**Figure 12:**
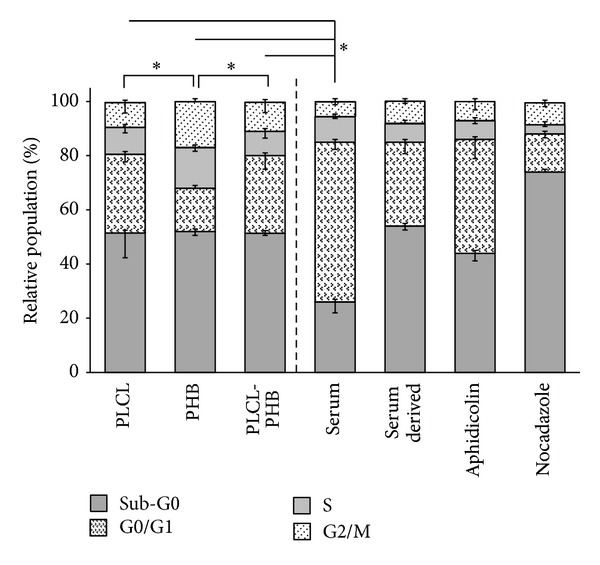
Distribution of cell populations in cell cycle phases, sub-G0, G0/G1, S and G2/M for OECs cultivated on electrospun scaffolds of PLCL, PHB and PLC/PHB (50 : 50 w/w) relative to cells in asynchronous growth; controls of cells in serum deprived media and with the additions of aphidicolin and nocodazole used to validate the experiment, (* = significance *P* < 0.05, *n* = 5).

Further evidence of changes in OEC health as a consequence of cultivation on the scaffolds was determined through analysis of their cell cycle (Figure 12). OECs in asynchronous growth exhibited a cell cycle profile with relative populations that consisted of cells in the sub-G0 (26 ± 4%), G0/G1 (59 ± 3%), S (9 ± 1%), and G1/M (6 ± 1%) phases. Conditions of serum deprivation maintained the cells in G0/G1 quiescent phase (31 ± 4%) while the addition of arresting agents, aphidicolin, and nocodazole maintained OECs in G0/G1 phase at 42 ± 7% and 14 ± 1%, respectively, thus validating the experiment (Figure 12). OECs cultivated on the scaffolds showed significant increases in the distribution of cell populations in the Sub-G0 phase, ca. 51 ± 3% compared to 26 ± 4% for those in asynchronous growth (*P* < 0.05, Figure 12). For OECs cultivated on the PLCL and the blended PLCL/PHB scaffolds, this increase in the Sub-G0 populations was accompanied by the same reduction in cells in the G0/G1 phase, (29 ± 3%). There were no significant differences in the OEC populations in the S and G2/M phases when cultivated on the PLCL and PLCL/PHB scaffolds compared to those in asynchronous growth.

In contrast to OECs cultivated on the PLCL based scaffolds, PHB appeared to promote the cell cycle, with significantly less OECs in the resting phase (G0/G1) compared to their PLCL based counterparts (16 ± 3%) and more in the synthesis (S, 15 ± 2%) and mitosis (G2/M, 17 ± 1%) phases (Figure 12). Thus, consistent with the other measurements of cell growth and health, the cell cycle analysis suggests a shift by the OECs to more proliferative growth in the presence of the biomaterial nanofibrous scaffolds. However, the similarity in cell phase distribution between the OECs grown on the scaffolds and those cultivated in serum-free growth media suggests cultivation to the point of media exhaustion. In this regard, the monomeric component of PHB, 3-HBA, is known to promote cell proliferation [34, 41].

Ahmed et al. reports that OECs cultivated on PHB films showed no significant variations in the distribution of cell cycle phases when compared to those cultivated under asynchronous conditions. Thus, the promotion of OEC cell cycle progression when cultivated on nanofibrous PHB scaffolds determined here supports the concept that electrospinning can enhance cell growth [30]. However, the results here are also consistent with the cell cycle behaviour for mesenchymal stem cells on PHB films, which varied significantly from their asynchronous growth control with a greater proportion of cells arresting in the S phase, confirming previous reports that cell response to biomaterials is lineage dependent [30].

## 4. Conclusions

Nanofibrous scaffolds of PLCL, PHB, and a range of their blends were successfully prepared by electrospinning. As the PLCL loading in PHB blends increased, the average fibre diameters in the electrospun scaffolds decreased significantly. Furthermore, the distribution of diameters was also reduced and shifted towards the lower diameter range. Similarly, the scaffolds increased in their flexibility with increasing PLCL loads. In contrast, PHB promoted the adhesion and growth of OECs compared to PLCL, but scaffolds produced from a 50 : 50 (% w/w) blend of the two showed enhanced cell proliferation. Analysis of cell health through membrane leakage, cell cycle progression, and apoptotic indices showed that the PLCL/PHB nanofibrous membrane promoted cell health and reduced necrosis. Thus, a combination of PHB with PLCL supported morphological changes when electrospun into nanofibrous scaffolds which, in turn, supported changes in hydrophilicity. These changes supported the attachment and proliferation of OECs without affecting the cell health. This study provides preliminary findings for further* in vivo* studies and investigation of electrospun PLCL/PHB as potential scaffolds for nerve repair.

## Figures and Tables

**Table 1:** Identification of peaks in FTIR spectra obtained from solubilised electrospun fibres of PHB, PLCL, and their blends.

PHB	O–H	C=O	CH–	C–O	O–H
Loading	Stretching		Group	Stretching	Bending (cm^−1^)
100	3502	1753	1273	—	—
75	3437	1723	1278	1057	1380
50	—	1735	1276	1095	1378
25	3442	1730	1288	974	1378
0	—	—	—	1056	1380
